# Physical Interaction Is Required in Social Buffering Induced by a Familiar Conspecific

**DOI:** 10.1038/srep39788

**Published:** 2016-12-23

**Authors:** Hou Liu, Ti-Fei Yuan

**Affiliations:** 1 School of psychology, Nanjing Normal University, Nanjing, 210097 China

## Abstract

In social animals, signals released from fearless conspecifics attenuate fear responses, namely social buffering. The presence of conspecific odor can suppress the expression of freezing response of conditioned mice. The present study investigated if physical social experience is required for this social buffering effect. The mice were exposed to donors, donor bedding (collected from cages of donors), or fresh bedding as control, respectively, for 10 days (1 hour daily) in prior to fear conditioning test. The fear expression test was examined in presence of donor bedding. The results showed that only the donor group mice showed reduced freezing time than the other two groups in the fear memory test. This phenomenon indicated that physical interaction might be required for the social buffering effect.

In social animals, presence of affiliated conspecifics influence fear responses. For instance, signals released from fearful conspecifics aggravated fear responses^1^. On the other hand, fearless conspecifics could release signals that attenuate fear responses at various levels, including behavioral, autonomic and neural levels^2^,^3^,^4^, namely social buffering. This buffering effect can be induced either by ‘pair-housing’ after a stressful event, or by ‘pair-exposure’ to an acute stressor with a conspecific animal^5^.

Social buffering is recognized as a multi-sensory modality effect, consisting of direct physical contact, visual observation^6^, and olfaction, for instance^7^,^8^. Notably, lesion of the main olfactory epithelium abolished this buffering effect^7^, indicating the importance of olfactory system underling the social buffering phenomenon. Interestingly, the suppression of the fear responses by a familiar conspecific was greater than an unfamiliar one^9^. This pointed out that the both the memory for odor identity (“what is the odor”?) and acquired value (“is the odor more rewarded”?, such as social familiarity) might be involved in this synthetic processing of the social buffering effect^10^.

Physical interaction is a crucial factor of individual recognition but not individual odor recognition. The prior physical interaction was required for hamster to discriminate different individuals in across-odor habituation^11^,^12^, but not in single-odor habituation tests^13^. These studies indicated that there are two classes of social odor learning in relation to distance-based response pattern^14^. In particular, different communication and behavior strategies are employed in the volatile distance or nose-contact distance^15^. In present study, we investigated that if the prior physical contact is required for the odor based social buffering effect for fear memory expression.

## Methods

### Animals

6-week-old experimentally naïve male wild-type C57BL/6 N mice were bred and kept in IVC. The room temperature was controlled at 24 ± 1 °C. Food and water were available ad libitum. 6 mice were assigned to singly housed donor group that was used to odorize the wood bedding. 17 animals were divided to social group (n = 6), odor+ group (n = 6) and odor− group (n = 5). 2 animals were housed in one cage, and cage mates were assigned to the same group. A donor would serve as a familiar donor for the subjects of social or odor+ group and as an unfamiliar donor for the subjects in odor− group.

Animal maintenance and use were performed in accordance with the National Technical Committee on Laboratory Animal Science of the Standardization Administration of China guidelines. All experimental procedures were approved by the Institution’s Animal Care and Use Committees of Nanjing Normal University, China.

### Odor Stimuli from donor cage

5 days prior to formal experiment, donor rats were given 600 cc of fresh wood bedding^16^,^17^. In continuous 10 days’ odor exposure, 200 cc samples were collected and served as the odorants for the exposure. These samples contained bedding, feces, urine, and mice food particles. Equal amount of fresh bedding was mixed into the remainder. The night before fear-expression day all bedding was collected and stored hermetically for following test.

**Figure 1:**
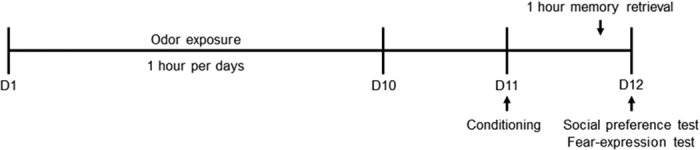
The procedure flow of the experiment.

Behavioral experiments included odor exposure phase, fear conditioning, memory retrieval, social novelty preference test and fear-expression test. All procedures took place during 10:00 to 14:00 (see Fig. 1).

### Odor exposure phase

Subjects were exposed to odorants daily for 1 hour during 10 days as previously described^18^. During exposure, subjects in social group were kept in cages which bottoms were covered by fresh wood bedding with donors together. Subjects in odor+ group and in odor− group were kept singly in cages which bottoms were covered by samples collected from cages of donors or fresh wood bedding, respectively.

### Fear conditioning

24 hours after the last exposure, mice were subjected for fear conditioning. Briefly, mice were placed in a conditioning chamber (rectangle, white) for 120 seconds before a 30 seconds’ tone stimulus (2900 Hz, 80 dB). An electrical foot shock (0.65 mA) was presented during the last 1 second of the tone presentation and co-terminates with the tone. Five trials of conditioning were separated by inter-trial intervals randomly range from 30 seconds to 150 seconds (on average 90 seconds). Following an additional stay for 60 second in the chamber, the animals were removed back to home cage^19^. Cage mates were manipulated simultaneously at all time.

### Memory retrieval

24 hours after the fear conditioning, subjects exposed to odorants for 1 hour to retrieve memory of odor. The manipulation of memory retrieval was the same as odor exposure. Social novelty preference test and fear-expression test following memory retrieval instantly.

### Social novelty preference test

**Figure 2:**
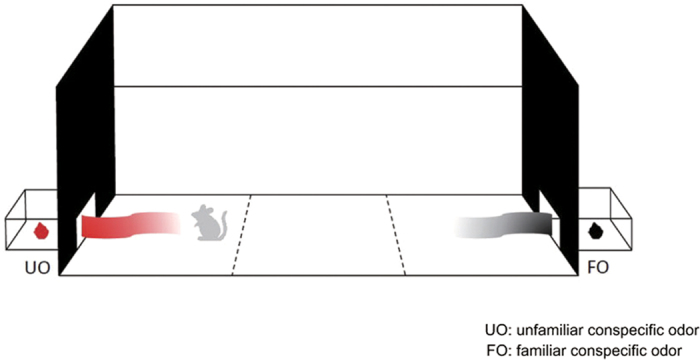
Bedding of donors (black cotton in picture) was placed in the side preferred previously, and bedding of unfamiliar conspecifics (red cotton in picture) were placed in another side.

Subjects were placed in the middle of a social chamber, with both sides connected with a small box. A piece of gauze separated the box from the chamber. In the first session, mice had a 5 minutes’ exploration in the chamber. Then bedding collected from the familiar donor cage (FO) and from cage of another unfamiliar mouse (UO) were placed into the two boxes respectively, followed by 10 minutes’ social exploration in the second session^20^. The FO was always placed in the box preferred previously. For odor− group, bedding collected from two unfamiliar mice was placed in two boxes randomly and a similar 10 minutes’ exploration would be delivered (see Fig. 2).

### Fear-expression test

Cued fear test was conducted in a chamber with a different context (triangle, black). The bedding stored hermetically was laid on the salver of the fear conditioning chamber. Testing was performed with the similar procedure as in the conditioning period but engaged 90 seconds’ intervals, without US^19^.

### Data analyses and statistical procedures

Social preference measures were taken of the amount of time spent in each side of chamber^20^. Preference scores were calculated by subtracting the time spent in FO from the time spent in UO for donor and odor+ groups, or by subtracting the time spent in left from the time spent in right for odor− group. The baselines in the first session were calculated for all subjects.

Freezing behaviors defined as the complete absence of any movement except for respiration and heartbeat lasting for longer than 2 second were timed during the testing session. The percentage of freezing time on or off tone presentation was recorded as CS+ or CS−, respectively.

Repeated measurement ANOVAs were employed in analyzing preference scores with the between-subjects factors group (social group, odor+ group, and odor− group) and the within-subjects factors time (pre, post). The same statistical test was used for freezing percentage with the between-subjects factors (social group, odor+ group, and odor− group) and the within-subjects factors cue (CS+, CS−). LSD Post hoc test was employed for pairwise comparisons. *p* < 0.05 was considered statistically significant.

## Results

### Social novelty preference test

**Figure 3:**
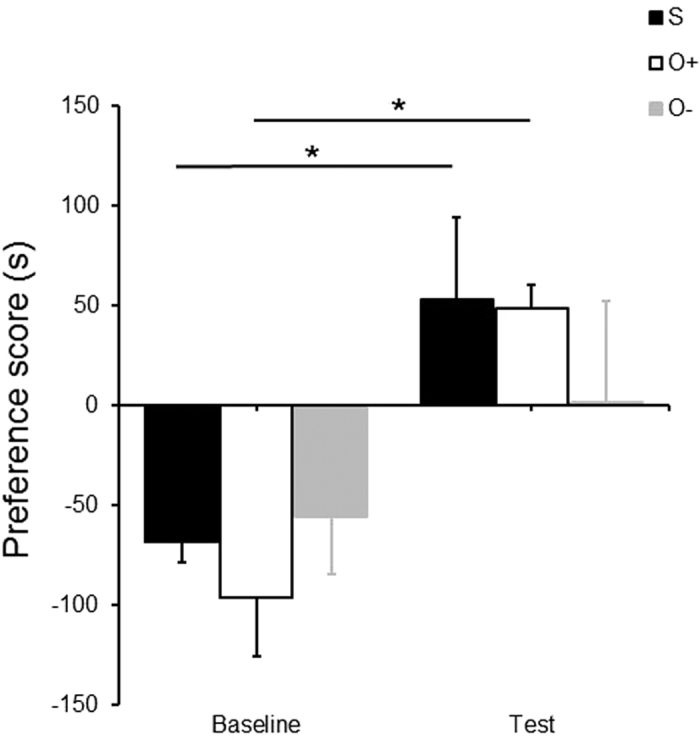
Preference (Post) to unfamiliar conspecific odor (UO) reversed prior position preference (Pre) in social (S) and odor+ groups (O+). But in odor− group (O−), there was no such reversal. *p < 0.05.

We compared preference for the side with an unfamiliar conspecific with baseline, and found a significant main effect of time (*F*_*1,13*_ = 15.501, *p* < 0.01, 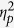  = 0.544). Simple main effect analysis showed that there was a significant effect of time in social group (*F*_*1,13*_ = 7.383, *p* < 0.05) and odor+ group (*F*_*1,13*_ = 8.836, *p* < 0.05), but not in odor− group (see Fig. 3).

### Fear-expression test

We tested whether there are some differences in freezing ratio between groups and conditions of cue (presence or absence). There was a significant main effect of cue (*F*_*1,14*_ = 67.830, *p* < 0.001, 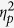  = 0.829) and a significant interaction of cue and groups (*F*_*2,14*_ = 7.219, *p* < 0.01, 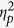  = 0.508), although no significant effect of groups.

**Figure 4:**
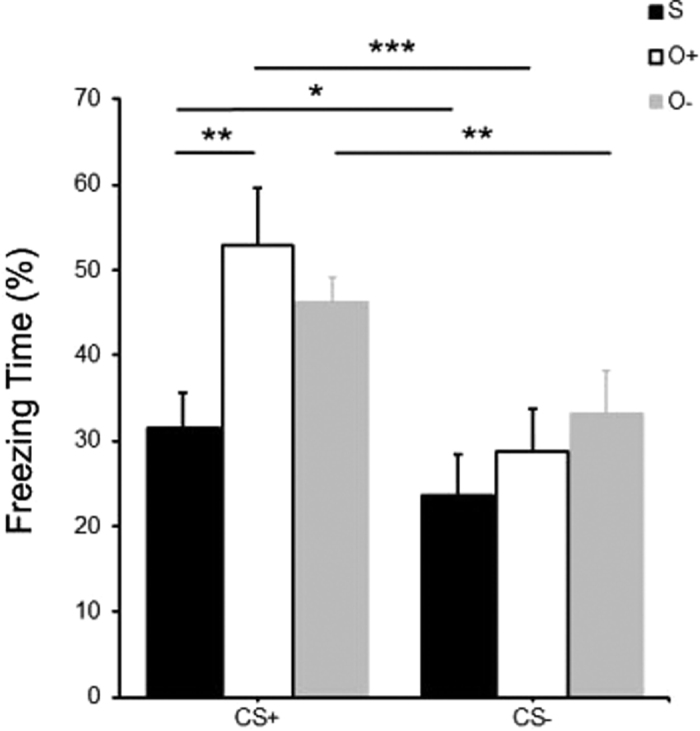
Tone cue presentation (CS+) induced freezing response of mice effectively. During presentation, subjects in social group (S) showed significant lower freezing percentage than odor+ group (O+) and odor− group (O−). But there was no difference between three groups without tone cue (CS−). *p < 0.05, **p < 0.01, ***p < 0.001, &0.05 < p < 0.1.

Simple main effect analysis showed that there was a significant effect of cue in social group (*F*_*1,14*_ = 6.539, *p* < 0.05), odor+ group (*F*_*1,13*_ = 61.552, *p* < 0.001), and odor− group (*F*_*1,14*_ = 15.639, *p* < 0.01). At CS+ level, there was a significant effect of groups (*F*_*2,14*_ = 4.839, *p* < 0.05). The LSD post hoc tests showed significant difference between social group and odor+ group (*p* < 0.01), and marginal significant difference between social group and odor− group (*p* = 0.065). But at CS− level, there was no significant effect of groups (see Fig. 4).

## Discussion

### Odor exposure improved the familiarity with the odor

In the first session, all groups spent equal time in each side. But social group and odor+ group spent more time in the UO later. The preferences indicated that UO was a novel social stimulus by comparison with FO, and drew mice’s attention to explore^11^,^12^,^13^,^20^,^21^. In other words, the FO became a familiar odor after 10-days odor exposure, no matter whether physical interaction took place during exposure or not. Consistent with this, subjects in odor− group did not show the preference.

### Odor exposure and physical interaction facilitated social buffering

All subjects showed increased freezing percentage on tone presentation, which proved that tone cue induced fear responses effectively. More importantly, at CS+ level, social group showed reduced freezing time than other groups. Because bedding used in test were collected from the same donor, the difference between groups should be due to the different manipulations during odor exposure.

Kiyokawa and his colleague^9^ found that a familiar conspecific is more effective than an unfamiliar conspecific for social buffering, as we have seen in this experiment. They assumed housing with a donor induced the plasticity in the MOB, enabling the subjects to perceive familiar odor more effectively. But some researches indicated that only odor exposure potentiated MOB response specificity^22^ but depressed response intensity^23^,^24^, which did not support the hypothesis. The present result that only odor exposure did not induce a greater social buffering in odor+ group also contradicted this assumption.

Olfaction is synthetic perception processing in which odors are initially encoded as ‘objects’^25^, and linked with specific biological significance^26^,^27^. Natural odors are also associated with experience-dependent significance or value^28^,^29^, which means delicious food or, in present study, a familiar conspecific. So an alternative hypothesis emerged that specific social value of a familiar conspecific was acquired by olfactory learning^30^, which a phenomenon was similar with that showed in previous researches^21^,^31^,^32^.

Both in the previous and present study, physical interaction played a required role for acquisition of social value. It was the prerequisite for individual recognition that physical contact was allowed in hamsters^11^,^12^,^13^, which could be an explanation for results that social group showed lower freezing percentage than other groups.

Social group showed considerable but not significant decline of freezing time when compared to odor− group. The reason might be a time lag detected in the odor− group, in which the max response sometimes appeared after the tone presentation. It should be noted that different from some researches^5^,^8^,^9^, only freezing behavior occurred during 30 seconds’ tone presentation was recorded as response to cue.

The other explanation for our finding is that bedding collected from cages of donors did not supplied odor as effective as donor itself. However, a significant preference to UO in odor+ group was found; and something similar to that was found in social groups. These facts indicated that olfactory learning took place in two groups equally. Besides, odor− group without donor odor exposure showed the same lever of freezing time as odor+ group. Moreover, fear test used bedding as odorants, which had been used as odorants for exposure in odor+ group, so social group did not have additional advantage due to consistency of learning and test.

To summarize, our result suggests that physical interaction is required in social buffering induced by a familiar conspecific.

## Additional Information

**How to cite this article**: Liu, H. and Yuan, T.-F. Physical Interaction Is Required in Social Buffering Induced by a Familiar Conspecific. *Sci. Rep.* **6**, 39788; doi: 10.1038/srep39788 (2016).

**Publisher's note:** Springer Nature remains neutral with regard to jurisdictional claims in published maps and institutional affiliations.
